# Transcriptome analysis of rumen epithelium and meta-transcriptome analysis of rumen epimural microbial community in young calves with feed induced acidosis

**DOI:** 10.1038/s41598-019-40375-2

**Published:** 2019-03-18

**Authors:** Wenli Li, Sonia Gelsinger, Andrea Edwards, Christina Riehle, Daniel Koch

**Affiliations:** 1 The Cell Wall Utilization and Biology Laboratory, US Dairy Forage Research Center, USDA ARS, Madison, WI 53706 USA; 2 Department of Dairy Science, University of Wisconsin-Madison, Madison, WI 53706 USA; 3 Department of Genetics, University of Wisconsin-Madison, Madison, WI 53706 USA; 4 Department of Computer Engineering, University of Wisconsin-Madison, Madison, WI 53706 USA

## Abstract

Many common management practices used to raise dairy calves while on milk and during weaning can cause rumen acidosis. Ruminal pH has long been used to identify ruminal acidosis. However, few attempts were undertaken to understand the role of prolonged ruminal acidosis on rumen microbial community or host health in young calves long after weaning. Thus, the molecular changes associated with prolonged rumen acidosis in post weaning young calves are largely unknown. In this study, we induced ruminal acidosis by feeding a highly processed, starch-rich diet to calves starting from one week of age through 16 weeks. Rumen epithelial tissues were collected at necropsy at 17 weeks of age. Transcriptome analyses on the rumen epithelium and meta-transcriptome analysis of rumen epimural microbial communities were carried out. Calves with induced ruminal acidosis showed significantly less weight gain over the course of the experiment, in addition to substantially lower ruminal pH in comparison to the control group. For rumen epithelial transcriptome, a total of 672 genes (fold-change, FC ≥ 1.5; adjusted-*p* ≤ 0.05) showed significant differential expression in comparison to control. Biological pathways impacted by these differentially expressed genes included cell signaling and morphogenesis, indicating the impact of ruminal acidosis on rumen epithelium development. rRNA read-based microbial classification indicated significant increase in abundance of several genera in calves with induced acidosis. Our study provides insight into host rumen transcriptome changes associated with prolonged acidosis in post weaning calves. Shifts in microbial species abundance are promising for microbial species-based biomarker development and artificial manipulation. Such knowledge provides a foundation for future more precise diagnosis and preventative management of rumen acidosis in dairy calves.

## Introduction

Ruminal acidosis is a well-recognized digestive disorder found in dairy cattle^1^. In order to maintain high milk yield, dairy cattle diets have become more nutrient-dense, containing highly fermentable carbohydrates. In some cases, this can lead to an accumulation of volatile fatty acids (VFAs) and reduced buffering capacity in the rumen^2,3^, lowering ruminal pH. Sub-acute ruminal acidosis (SARA) is defined as a metabolic disorder caused by the ingestion of diets rich in rapidly fermentable carbohydrates with insufficient amount of fiber required for efficient rumen buffering, leading to an overall reduction in ruminal pH^4,5^. Symptoms of the disease include rumen epithelial damage^6–8^, inflammation^9^, laminitis^10^, decreased dry matter intake^2^, reduced *in situ* fiber degradation^11^, and liver abscesses^12,13^. Field studies documenting the prevalence of SARA in mature dairy cows reported incidence rates as high as 19% of the total herd, and up to 26% in mid-lactation cows^14^. The direct results of SARA-induced digestive and metabolic disfunction include milk yield reduction, decreased production efficiency, premature culling and increased mortality. Consequently, the estimated economic loss attributed to SARA is between $500 million to $1 billion annually^15^, making SARA one of the most important nutritional diseases in dairy cattle.

Though low ruminal pH has been used for the diagnosis of SARA, there is a significant discrepancy in the literature regarding the exact threshold of ruminal pH to be used. For example, it has been reported that SARA was determined when ruminal pH dropped below threshold values of 5.5^14,16^ 5.6^17^ (on average of 2.2–3.6 hours/day), 5.8^18^, and 6^19^. Some studies suggested that several episodes, during which the ruminal pH remained low (below 5.6 or 5.8) for longer than 3 or 5 hours per day, were a good indicator of SARA^20^. Recent studies that measured ruminal pH in young calves with acidosis reported mean pH values between 5.5 and 4.1 across various dietary treatments in the weeks surrounding weaning^21–24^. Such a range of variation in ruminal pH used to determine ruminal acidosis suggest a need to develop other tools/biomarkers that will facilitate the precise diagnosis and preventative management.

The rumen is not fully functional at birth and must increase in size, morphology and function in order to provide sufficient protein and energy to the host at the time of weaning, which occurs at 8 weeks of age in most dairy calves^25^. The production of VFAs, especially butyric acid, a by-product of starch fermentation in the rumen, is the primary stimulant required for rumen tissue development^26^. This finding has led to an emphasis on feeding highly fermentable grain mixes to calves to stimulate rumen development, thereby allowing calves to be weaned at an earlier age. Although the consumption of starter feed may seem beneficial to rumen development, calves fed starch sources during the weaning transition exhibited increased VFA production, leading to decreased ruminal pH^27^. Immunosuppression (caused by translocation of endotoxin into the systemic circulation^28^) and inflammation have also been associated with the depression of ruminal pH^2,4^. Thus, the high fermentability of the feed along with the lack of tissue development required for efficient VFA absorption might contribute to ruminal acidosis in young calves.

The composition and functions of the rumen microbiota have been linked to rumen acidosis^29,30^. Reduction in bacterial diversity, with predominant phyla in *Firmicutes*, *Bacteroidetes*, and all subgroups of *Proteobacteria*, has been observed among the cattle with SARA^30,31^. Young calves that have not been adapted to high grain diets are particularly susceptible to acute ruminal acidosis^32^. This is most likely due to the lack of a well-developed, viable population of rumen bacteria that can efficiently utilize lactic acid. Changes in the timing and availability of dietary substrate composition have also been linked to the changes in the rumen bacterial community function and composition^33–36^. Analyses into the alteration and population dynamics of the ruminal microbiome in response to feed induced acidosis may help identify microbial species-based biomarkers. Further application of such biomarkers holds the potential for a more precise diagnosis of SARA and subsequent preventative treatment of SARA.

The rumen epithelial tissue has multiple metabolic roles vital to the health of a cow. These include nutrient absorption, metabolism, pH regulation, and immune and barrier function^37–39^. Despite these important functional roles, ruminal epithelial cells are vulnerable to acidosis, which typically leads to ruminal parakeratosis, erosion, and ulceration of the ruminal epithelium^40^. Rapid fermentation caused pH depression has been linked to the impairment of barrier function in the gut^41^. Few studies have evaluated the rumen microbial and transcriptome changes of the rumen epithelium related to high-grain or high-fiber diets during the calf weaning transition^42,43^. However, the effects of feed-induced ruminal acidosis on calf development and health long after weaning has not been investigated. Specifically, little is known about the impacts of feed-induced acidosis on rumen epithelial transcriptome and its associated microbial population composition in calves well after weaning. In this study, we used a highly-processed, starch-rich feed to induce ruminal acidosis in newborn bull calves beginning at one week of age through 16 weeks. At 17 weeks of age, rumen epithelial tissues were collected after sacrifice, followed by rumen epithelial tissue transcriptome analysis and rRNA-based microbial classification analysis of the rumen epimural community. We hypothesized that significant changes in rumen epithelial transcriptome and its associated epimural microbial community would be associated with the feed-induced acidosis.

## Results

### Starter intake, growth, ruminal pH and rumen volume in the treated and control groups

**Figure 1:**
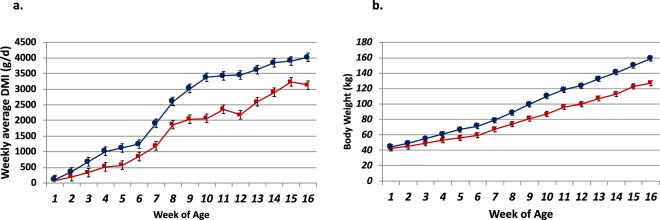
Average weekly starter intake and weight gains in the treated and control grops. (**a)** Average (with s.e.) weekly starter intake in the treated and control groups. (**b)** Average (with s.e.) body weight gains in the treated and control groups. The red lines are treatment and the blue lines are control.

**Figure 2:**
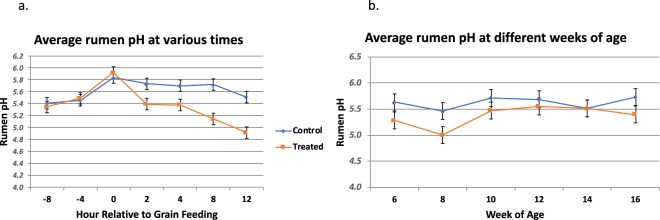
Average rumen pH for the treated and control group. (**a)** Average rumen pH across all weeks of age at each sampling time. Calves receiving the treatment diet had significantly lower rumen pH (p < 0.05) than control calves beginning 2 hours post-feeding through 12 hours post-feeding. (**b)** Average rumen pH across all sampling times for each week of age.

The clear difference between treatments in dry matter intake and body weight started at week four and five, respectively. Compared with the control group, a lower starter intake was observed for calves in the treated group throughout the experiment (*p-value* < 0.001) (Fig. 1a). Consistently, we observed significantly lower body weight for treated calves (*p-value* < 0.001) across all weeks (Fig. 1b). Significantly lower ruminal pH was observed in the treated group using data collected throughout all weeks and all sampling times (*p-value* < 0.005) Fig. 2a,b). The lowest mean rumen pH was observed during the week of weaning. (Fig. 2b). The estimated rumen volume of treated group was 12 ± 1.2 (s.e.) L, which was significantly smaller (p ≪ 0.05) than the control group (17 ± 2.3 (s.e.) L).

### RNA quality and sequencing reads alignment for rumen papilla tissue

For rumen papilla tissue, the extracted RNA samples were of high quality, with the average RNA integrity number (RIN) of 9.01 ± 0.25 (s.e.). Total number of RNA sequencing raw-reads ranged from 69 M to 81 M, with an average of 76.8 M ± 1.29 M (s.e.). The total number of expressed genes ranged from 9,677 to 11,144 (Fragments Per Kilobase of transcript per Million mapped reads (FPKM), cutoff >=1) (Supplemental Table 1). All samples had similar distribution of gene expression using the four FPKM brackets, with majority of the genes expressed in the range of 0.2 to 15 FPKMs.

### Rumen epithelial transcriptome changes between calves fed with Treated (high-starch) and Control (low-starch) diets

**Figure 3:**
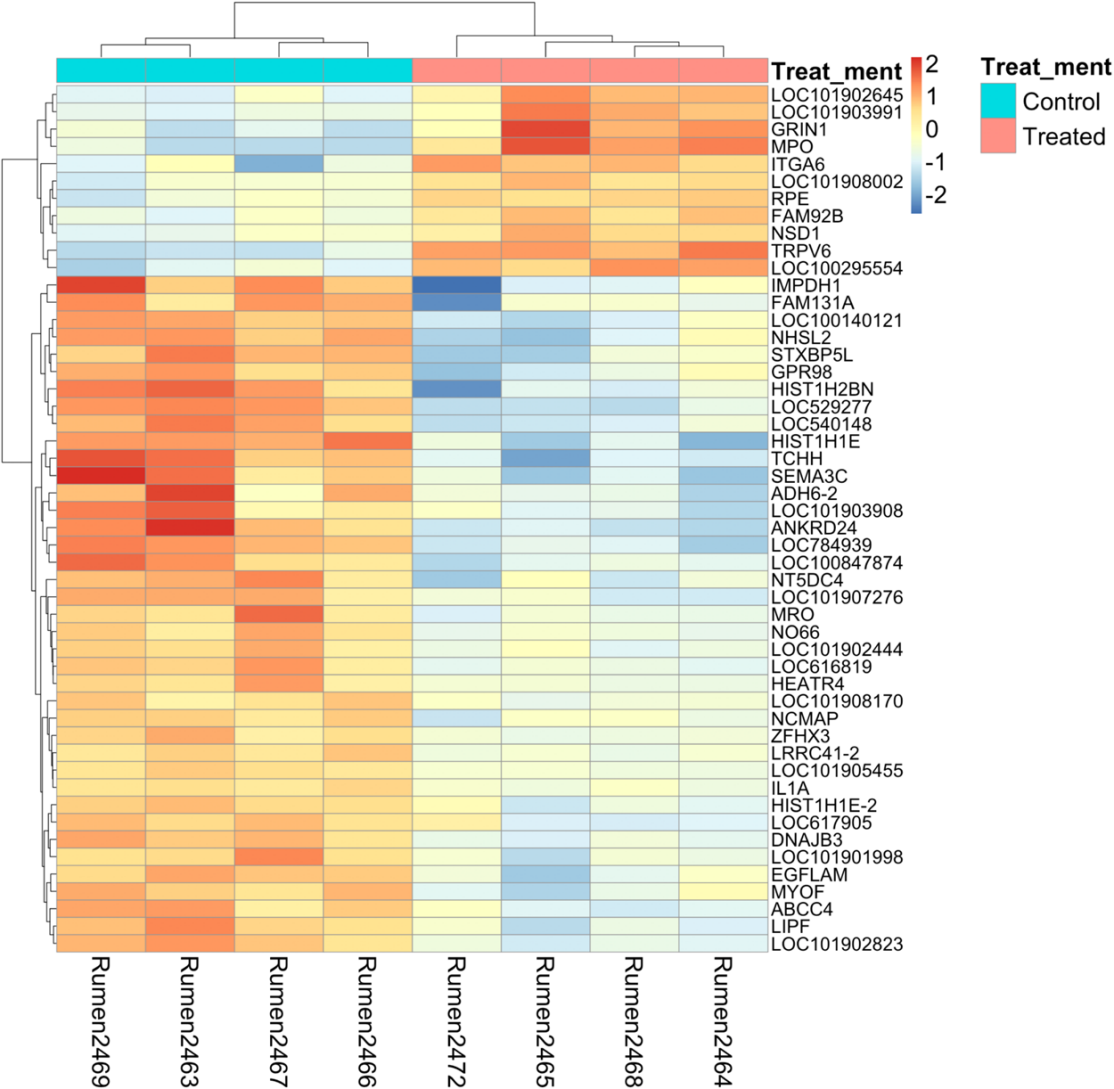
Clustering heat-map of top 50 most significant differentially expressed genes between the treated and control groups.

**Figure 4:**
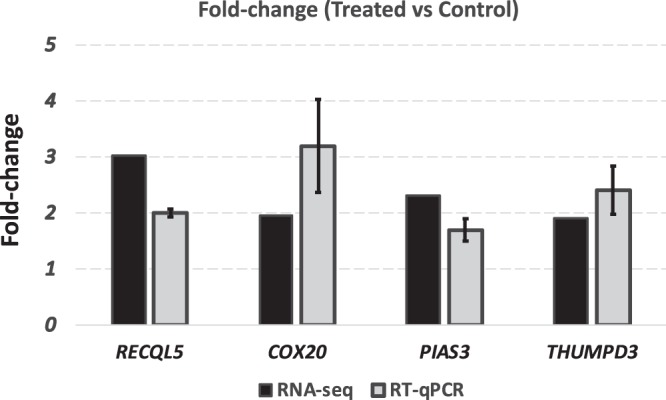
RT-qPCR confirmation of four differentially expressed genes identified by RNA-seq. Fold-change (Treated vs control) of target genes were calculated by both RNA-seq and RT-qPCR methods.

Between the treatment groups, a total of 672 DEGs were identified (Supplemental Table 2), with the top 50 most significant DEGs clearly separating the two groups of animals (Fig. 3). All four differentially expressed genes were successfully confirmed by RT-qPCR method (Fig. 4). Across all samples, 34 mostly abundantly expressed genes were identified, and these genes were enriched in microtubule organization (48 it should be 18 genes, *p-value* ≪ 0.00001). Among the top 1% most highly expressed genes between the control and treated group, 12 genes were uniquely expressed in the treated group (*COX5B*, *KRT78*, *KRT15*, *ATP5I*, *ATP5L*, *ATP5G2*, *COX8B*, *COX8A*, *UBC*, *DSP*, *ITM2B*, and *C10H15orf48*). GO pathway analysis indicated that these genes were significantly enriched in hydrogen ion transmembrane transport (GO:BP~GO:1902600; 5 genes; *p-value* ≪ 0.00001).

For the top 50 most differentially expressed genes, a significant enrichment in GO:CC~actin cytoskeleton was identified. Using either the list of up-regulated genes or the list of down-regulated genes, the most significant pathway identified is GO:CC~nucleus (*p-*value ≪ 0.00001). GO pathways analysis using the combined list of DEGs indicated the pathways involving cell division and growth. They include membrane-bounded organelle (GO:0043227; 296 genes; *p-*value ≪ 0.0001), cytoplasm (GO:0071840; 259 genes; *p-*value ≪ 0.0001), cellular component organization or biogenesis (GO:0043229; 293 genes; *p*-value ≪ 0.0001), nucleus synthesis (GO:0005634;165 genes; *p-*value ≪ 0.0001), and membrane-enclosed lumen (GO:0031974; 99 gene; *p-*value ≪ 0.001). Additionally, a significant portion of the DEGs were involved in cellular metabolic processes, including cellular nitrogen compound metabolic process (GO:0034641; 151 genes; *p-*value < 0.001), cellular lipid metabolic process (GO:0044255; 27 genes; *p-value* < 0.05) and glycoprotein synthesis (GO:0009101; 86 genes; *p-value* < 0.05).

### Results of taxonomic classification of rumen epithelial microbial community and the correlation of rumen mRNA and rumen microbial rRNA expression

**Figure 5:**
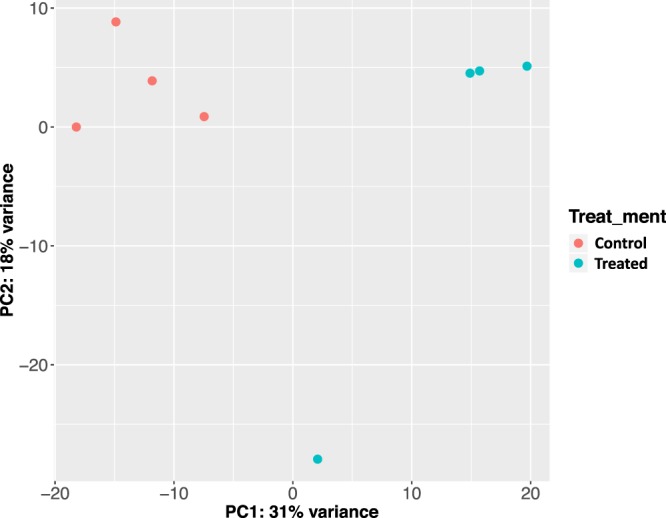
PCA plot of rRNA samples using normalized, genus level read-counts. Control and treated animals separate along PC1 and PC2, accounting 49% of the overall differences. Rumen microbial rRNA reads were obtained by rumen papillae tissue RNA-seq. Genus classification was done using Kraken.

**Figure 6:**
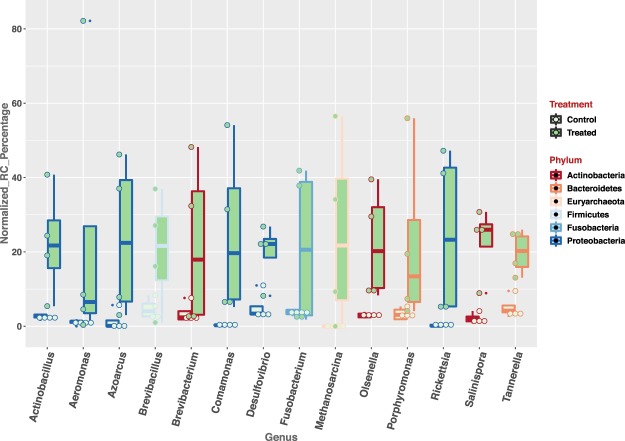
The abundance of 14 genera is significantly higher in treated group (*p*-value < 0.05), in comparison to the control group. rRNA sequencing reads mapped to each genera by Kraken were used to calculate the normalized read-counts.

**Figure 7:**
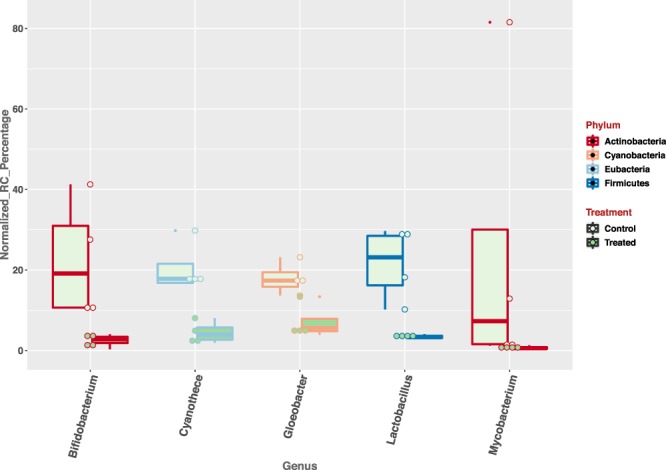
The abundance of 5 genera is significantly higher in the control group (*p*-value < 0.05), in comparison to the treated group. rRNA sequencing reads mapped to each genera by Kraken were used to calculate the normalized read-counts.

The average number of rRNA reads used for rumen wall microbial classification is 1.4 M ± 0.34 M (mean ± s.e.). A high classification rate was achieved for each sample, with the mean Kraken classification rate as 99.39 ± 0.13 (mean ± s.e.). Using genus-level, normalized read-counts, a clear separation of treated and control groups were observed along PC1 and PC2, totaling 49% of the observed variations (Fig. 5). By comparing the genus-level microbial abundance between the treated and control groups, we identified 14 genera with significant increase in the treated group (Fig. 6) (p-value < 0.05 and normalized read-count >=100); and 5 genera with significant increase in the control group (Fig. 7) (p*-*value < 0.05 and normalized read-count >=100). Among the genera that showed significant increase in treated group, majority of them are gram-negative bacteria (Supplemental Table 3); while gram-positive bacteria dominated the genera identified with significant increase in control group (Supplemental Table 4).

We identified 50 differentially expressed genes with significant association with rumen microbial rRNA expression at the genus level (*p-value* < 0.0001). A significant enrichment in membrane-bounded organelle (GO:0043227; 26 genes; *p*-*value* < 0.05) was identified. Among these, 14 genes were directly involved in membrane biogenesis (Supplemental Table 5). A total of 15 genera were identified with significant positive correlation in rRNA expression with these genes.

## Discussion

### Decreased ruminal pH, feed intake and overall weight gain in treated (high-starch) calves

Reduced ruminal pH has been reported in both goats^44^ and lactating dairy cattle^45^ consuming feeds high in dietary-starch content. Previous studies where rumen pH was recorded demonstrate a drop in pH at the time of weaning^21,23,24,46^. In our study, we also observed the lowest mean rumen pH during the week of weaning, indicating the calves experience the greatest acidosis challenge during weaning (with ruminal pH < 5.5). Our data is the first that we are aware of documenting changes in rumen pH post-weaning. Suarez-Mena *et al*.^24^ demonstrated that larger particle size (lower degree of processing) reduced the risk of acidosis in calves. Similarly, calves in the current experiment with the less-processed diet (control) experienced higher average rumen pH values compared to calves fed a more processed diet (treatment). Throughout the experiment, significantly lower ruminal pH was observed in the treated group. Additionally, the calves in the treated group had significantly lower starter intake and weight-gain over the course of the experiment. Ruminal epithelium is critical for nutrient absorption and transportation^47,48^, in addition to short-chain fatty acid (SCFA) metabolism^49^. Favorable development of rumen epithelial structure is associated with the absorption of VFA^49,50^. The development of rumen papillae was stimulated in calves consuming increased amount of concentrated as reported by Zitnan *et al*.^51^. However, when the amount of VFA exceeds the absorbing ability of rumen papillae, the excessive amount of VFA will lead to substantially reduced ruminal pH and the destruction of ruminal epithelium. Increased dietary starch reportedly played a role in causing SARA, and prolonged SARA then led to damage of the rumen epithelium^4,47^. Consequently, loss of appetite was reported as one of the typical clinical symptom of ruminal acidosis both in young calves^52^, and dairy cattle^20^. Taken together, we reason that the starch-rich diet fed to the treated group impaired the normal functionality of the rumen epithelium. Resultantly, the rate and ability of the rumen to absorb nutrients is hampered by the damaged rumen epithelium caused by ruminal wall lesions and the condition of ruminal parakeratosis^7,8^, as reflected by the reduced starter-intake and depressed weight gain in treated calves. As a follow-up study, histology analysis of rumen epithelium collected from the treated and control groups will provide further insights into the degree and prevalence of high-starch diet-induced rumen papillae damage.

### Acidosis-inducing diet on most highly expressed genes in rumen epithelial transcriptome

For the top 1%, most highly expressed genes between control and treated groups, 12 of them are uniquely expressed in the treated group. These genes were significantly enriched in the biological process of hydrogen ion transmembrane transport. In the stomach, hydrogen ions (customarily used to represent a proton) are moved through the membrane of a parietal cell and into the duct of a gastric gland via a proton pump known as H^+^/K^+^ ATPase^53,54^. In the rumen, during the process of fermentation of organic-matter in feedstuff, hydrogen ions are removed from the rumen by absorption across the rumen wall or passage through the digestive tract. As a major source of buffer, saliva facilitates the removal of hydrogen ions from rumen^55^. If this removal process is not efficient, it will lead to hydrogen ion accumulation, pH (a measure of hydrogen ion concentration) falls. Laarman and Oba^27^ reported that calves fed starch sources during the weaning transition showed increased VFA and lactic acid production, leading to significant decreases in ruminal pH. Similarly, previously published work suggested that a diet rich in fermentable-carbohydrates can lead to a dramatic drop in ruminal pH^4^. In our study, the treated calves were fed a starch-rich, highly-processed, rapidly-fermentable diet, and we observed consistently lower ruminal pH in the treated group across all sampling points. Additionally, it has been clearly demonstrated that both fermentation and neutralization processes occurring in the rumen directly contributed to the occurrence of SARA^55,56^. Taken together, our findings suggested that, in the treated group, the rumen epithelium can metabolically adapt to a starch-rich diet at the molecular level, as indicated by the enrichment of most highly expressed genes in hydrogen ion transport.

The most highly expressed genes identified in our study may provide a new remedy to help control or mediate diet-associated ruminal acidosis. Feeding a diet enriched with fermentable-carbohydrates may help the energy and nutrient requirements of the calves. On the other hand, it is equally important to avoid digestive and systemic metabolic disturbances resultant from such diets. Development of feeding strategies and the design of feed rations that help the digestive health have been a focus of research in cattle. For example, in pre-weaned calves, addition of forages to the calf starter has been shown to increase ruminal pH in pre-weaned calves^43^. In mature cows, inclusion of dietary forage NDF promotes chewing, which in turn stimulates flow of alkaline saliva^55,56^. Interestingly, in human clinical pharmacology, Pantoprazole is commonly prescribed to patients with gastric acid reflux^57–59^. Pantoprazole is a proton pump inhibitor, which blocks the transport of hydrogen ion into the gastric lumen. Based on our findings, it might be useful to add proton pump inhibitor to the feed to help reduce the side-effect associated with ruminal acidosis. However, whether adding a proton pump inhibitor as a feed additive is as efficacious for maintaining production efficiency and gut-health as the fiber-rich counterparts has been yet to be studied. Additionally, the downstream nutritional impact of the use of Pantoprazole as a feed addition spanning the whole food chain requires careful evaluations. Another potentially fruitful path to pursue would be to perform targeted gene knock-out studies on the genes involved in the hydrogen ion transport using a mouse model. Further functional follow up using either knock-out or CRISPR gene editing technology on the host would most likely yield meaningful insights into host-genetics based management tools for feed-induced acidosis. Consequently, the overall impact of CRISPR gene editing aiming to improve cattle gut health requires extensive assessment and validation before they enter the food chain.

### Treated (High-starch) diet caused epithelial remodeling captured at the transcriptome level

In our study, the high-starch diet induced acidosis in the treated calves in comparison to the control group. Using the list of differentially expressed genes (DEGs) between the two treatments, the most significant GO terms identified in our study include GO:CC~actin cytoskeleton (using the top 50 most differentially expressed genes), and the pathways involved in cell division and growth (for both the up-regulated and down-regulated genes identified in treated group in comparison to the control). In ruminants, the effect of fermentable-carbohydrates and the resulting SCFA on epithelial cell division in the digestive tract have long been reported^60,61^. SCFA concentrations can rapidly stimulate growth in ruminal epithelium proliferation^60,62^ and morphogenesis^63^. However, diet-induced acidosis can lead to parakeratosis (thickening of the stratum cornea of the rumen mucosa), which can then severely compromise SCFA absorption. Using a high-grain diet induced acidosis model, Steele *et al*.^6^ reported a significant reduction of the total depth of epithelium, along with significant reduction of depth in the stratum basal, spinosum, and granulosum layers of the rumen epithelium. Specifically, Steele and co-authors reported large spaces between cells and deterioration of cellular junctions.

Our transcriptome data suggested that the rumen epithelium underwent an extensive remodeling at the molecular level, mostly involving the genes contributing to cytoskeleton and microtubule formation and arrangement. Microtubules are filamentous polymers that can grow, shrink or remain stable^64^. As a vital component of the cell, microtubules play critical roles in cell division, cell polarity and motility, cell signaling and intracellular transport^65,66^. The cytoskeleton formed by microtubules is essential to the morphogenesis of an organism’s development^67,68^. Most interestingly, actin cytoskeleton was previously identified as one of the most affected pathways after gene ontology analysis of DEGs in ruminal tissue from cows fed with low- or high-concentrate diets^69^. Consistent with our findings, several other studies also reported diet-induced morphological alterations of the ruminal epithelium^62,70–72^.

### Other biological processes impacted by the high-starch (Treated) diet induced acidosis

Several other biological pathways involved in cellular metabolic processes were also identified in our study. They include nitrogen compound metabolic process, lipid and glycoprotein synthesis. Ruminants are known for their unique ability to fix nonprotein nitrogen (NPN) into protein^73,74^. And this ability is enabled by the microbial synthesis in the rumen, which provides most of the protein used by ruminants. Ruminal acidosis limits the amount of readily fermented carbohydrates that can be utilized by the rumen microbes for microbial protein formation. The Cornell model^75^ predicts the reduced formation of microbial protein per unit of fermented energy when ruminal pH falls below 6.2. Furthermore, there was a reported negative linear relationship between microbial protein yield and the length of time when ruminal pH was at or below 5.4^76^. Thus, protein synthesis of the ruminal microorganism is directly related to the nitrogen metabolism in the rumen. And this is manifested by rumen epithelial DEGs enriched in the pathway of nitrogen compound metabolic process

Glycoprotein synthesis is known for its relation to the inflammatory response^77,78^. In cattle affected by SARA, an acute phase response (APR) was typically observed. APR is a multifactorial, innate immune and metabolic response of the host to eliminate agent(s) causing the disturbance, and to bring back the normal homeostasis^79^. The direct result of APR is the increase of a variety of glycoproteins in the serum^77^ A dramatic rise of acute phase proteins (APPs), serum amyloid A (SAA) and haptoglobin (Hp) in cattle peripheral blood has previously been established as biomarkers in cattle ruminal acidosis^17,80^. Of note, these two APPs also showed a four-fold expression increase in liver tissues collected from the treated calves compared to the control in our study (data not shown). During the acidosis challenge, rumen is at the fore-front of the inflammatory response. However, it is likely that the inflammatory response related genes may not show a correlated response in periphery blood. The next step would be to identify other APR associated glycoproteins that have consistent mRNA expression profile in both the rumen and peripheral blood, or in any other readily accessible tissue types. The identification of such mRNA transcripts will be of great significance to the development of mRNA-based biomarker for early prognosis of ruminal acidosis.

### Microbial population changes resultant from high-starch diet induced acidosis

A surplus of easily fermented carbohydrates coupled with low ruminal pH imposes a challenge to the rumen microbes^81^. The epimural microbial community is at the direct interface between the host and rumen content. Rumen epimural microbial community is distinct from those in the rumen content^82,83^, and has a multitude of physiologically important functions, including the scavenging of oxygen, the recycling of epithelial tissue and hydrolysis of urea^84–86^. In our study, for genera that showed significant increase in the treated group, two of them, *Olsenella* and *Desulfovibrio*, showed more than 4-fold rise in the treated group compared to the control group. This finding is consistent with a previous study in beef cattle, where both the genera were reported with a significant increase in ruminal acidosis^87^. Previous studies also indicated that SARA associated rumen microbial changes were enriched by the increases of gram negative bacteria (GNB)^9,36,81^. Similarly, in our findings, we observed that the GNB made up the majority of the genera showing significant increase in the treated group^9^. During SARA, depression of ruminal pH increases the lysis of Gram-negative bacteria^17^. Along with the increase of different genera of GNB, the lysis of the GNB leads to the dramatic increase of lipopolysaccharide (LPS) in the ruminal fluid^4,17,88^. As part of the cell-wall component of all GNB bacteria, LPS is a strong pro-inflammatory molecule and commonly known as endotoxin. Feeding dairy cattle a diet with a high percentage of grain concentrate has been consistently associated with increases of endotoxin^89,90^. In the absence of tissue lesion, depressed ruminal pH may increase the permeability of the rumen epithelium through reduced organization and thickness of the epithelium^6^. During this process, the endotoxin can be translocated from the rumen into the blood stream, causing an inflammatory response^91^. Previous work has shown that a significant portion of the variation in the response to rumen endotoxin was attributed to the individual animal’s inherent genomic profile^89^. Thus, under the ever growing pressure for high milk and protein production, there is an increasing need for nutrigenomics that focuses on delineating the genomic attributes and molecular mechanisms associated with the superior absorption and metabolic capability of the rumen epithelium while under a high-energy diet enriched with readily fermentable-carbohydrates.

*Fusobacterium* was another significantly increased genus observed in the treated group. Once erosion happens in the rumen epithelium during the process of rumen acidosis, the microbes in the rumen can translocate into the bloodstream, causing liver abscesses^13,63^. In particular, *Fusobacterium necrophorum* was reported as the primary etiologic microbe that invades the liver and causes abscesses in cattle^92^. As a rumen wall aerotolerant anaerobe, this organism’s role is to degrade feed, metabolize lactic acid and epithelial proteins. *Fusobacterium necrophorum* is also an opportunistic pathogen since its abundance is higher in grain-fed than forage-fed cattle^93^. Furthermore, *F*. *necrophorum* is frequently isolated from the ruminal wall, exhibiting parakeratosis and prolonged SARA, and less frequently from unaffected ruminal epithelium^94,95^. For future studies, it would be of great interest to investigate any signs of the liver abscesses in calves with feed-induced acidosis, and follow up with sequencing based investigation not only to confirm the presence of *Fusobacterium necrophorum*, but also to identify any other novel, abscesses-causing microbes in the liver. Such studies will most likely enable the discovery of acidosis-specific microbes that cause liver abscesses. The knowledge gained from these studies will also help more effective treatment of liver abscesses accompanied by ruminal acidosis.

Our study suggested a direct interaction between rumen epimural microbes and the host rumen tissues, as amplified by the identification of 15 microbial genera with significant positive correlation to host rumen mRNA gene expression profile. A significant portion of the affected genes are involved in membrane-bounded organelle, indicating the impact of feed induced microbial population changes on the rumen epithelium development. These findings pointed out the potential development of these 15 microbial genera into diagnostic biomarkers to facilitate the monitor and prevention of ruminal acidosis through feed management.

### Future perspectives

Our study represents a snap-shot of the host rumen epithelial transcriptome changes in response to the acidosis induced by a starch-rich diet in young calves. Results from this study indicated that a starch-rich, diet-induced ruminal acidosis is accompanied by significant rumen epithelial transcriptome changes. Furthermore, significant changes in microbial rRNA production in the rumen epithelium were observed. For future follow-up studies, comparative analysis spanning several time points in the young calf’s life will provide more quantitative measures regarding how and when certain transcripts in the host are affected by the feed-induced acidosis. Most interestingly, the identification of host mRNA transcripts or microbial rRNA transcripts that show consistent expression profile in other easily accessible tissue types deserve further efforts. Knowledge obtained through these studies will most likely facilitate the development of non-invasive, host mRNA- or microbial rRNA-based biomarkers. The identified biomarkers are likely to increase the precision in the prognosis, prevention and management of ruminal acidosis. Additionally, similar studies in feeding trials aimed at improving the productivity and performance of ruminants while maintaining optimal gut health hold a great potential to the development of precision ruminant feed. Such optimized feed design will meet the host ruminant’s needs at both the nutrient and production levels. More importantly, the optimized feed will facilitate the improved balances between the host’s metabolism and gut microbial ecology.

## Material and Methods

### Ethics statement and animal care

All animal procedures were reviewed and approved by the University of Wisconsin – Madison Institutional Animal Care and Use Committee (IACUC #A005848). All animals involved in this study were fed and watered according to the herd standard practices used at the USDA Dairy Forage Research Center farm throughout the experiment.

Eleven Holstein bull calves born at the Marshfield Agricultural Research Station (Marshfield, WI) between June 17 and July 5, 2017 were used for this experiment. Each calf was removed from their dam and received 3.79 L of colostrum within 3 hours of birth. Additional feedings of colostrum were offered until 48 h of age. Calves were included in the trial when serum total protein concentration (STP) > 5.5 g/dL. After 48 h, 1.90 L (227 g dry-matter) of milk replacer (22% CP, 20% fat; Land O’ Lakes, Inc., Arden Hills, MN) was offered via nipple bottle at 0700 and 1900 for 6 weeks and then at 0700 only for 7 d. Calves were weaned at 8 weeks of age. Calves were housed in individual calf hutches (4.8 sq. m/calf) from birth to 8 weeks and then divided into larger hutches (5.0 sq. m/calf) through 16 weeks. Water was provided ad libitum for the duration of the study.

### Study Design

Two grain starter diets were offered to the calves: the treated and the control. The high-starch diet (Treated) (A; pelleted, 42.7% starch, 15.1% neutral detergent fiber, (NDF)) was designed to induce rumen acidosis. The low-starch diet (Control) (B; texturized, 35.3% starch, 25.3% NDF) was designed to blunt rumen acidosis. The diet composition is reported in Gelsinger *et al*.^96^. Treatments were randomly assigned and offered to calves beginning at 1 week of age (6.6 ± 3.4 d). A measured amount of starter was offered daily at 0800 and refusals were determined daily. Calves were allowed *ad libitum* access to their assigned starter for the duration of the trial up to 4500 g/d. Rumen pH values were measured 7 times in a single day every other week beginning at week 6 through week 16. On a given day, rumen pH values were measured in the hours before feeding and after feeding. Rumen pH was measured by manually inserting a pH probe into the rumen via a rumen canula. These time-points were: −8, −4, 0, 2, 4, 8 and 12 hours relative to grain feeding. Starter intake and body weight were measured for all calves every week, beginning at week one through week 16. Rumen volume was estimated by Cobalt ethylenediamine tetraacetic acid (Co-EDTA) method^97^. Calves were euthanized through Stunning with a captive bolt at 17 weeks of age at the Marshfield Agricultural Research Station, United States Department of Agriculture (USDA) facility for tissue collection.

The control group has five calves and the treated group has six calves. A power analysis was performed using rumen pH as the criteria of interest. Assuming a power of 0.9 and alpha value 0.05, a minimum of 5 calves were needed per treatment. The control group has five calves and the treated group has six calves.

### Calf rumen epithelium tissue collection

For each treatment group, four randomly selected calves were subjected to rumen papillae tissue collection followed by RNA sequencing. These tissues were collected immediately after animal euthanasia. Papilla tissues were taken from same location at the cranial ventral rumen wall. Upon collection, tissues were rinsed in PBS to remove feed particles if present, and cut with sterilized scalpels into 4–5 mm^2^ fragments and put into Eppendorf safe-lock tubes (Eppendorf North America, Hauppauge, NY). Collected tissues were flash frozen in liquid nitrogen and stored at −80 °C for long-term storage.

### RNA extraction, quantification and whole transcriptome sequencing

Collected rumen papillae issues were homogenized into fine powders in liquid nitrogen using a mortar and pestle. RNAs were extracted following the miRNeasy protocol with a QIAcube instrument (Qiagen US). The quality of extracted RNAs was assessed using Bioanalyzer RNA 6000 nano kit (Agilent Technologies, US). RNA samples with RNA integrity number (RIN) value ≥ 8 were pursued for RNA quantification using Qbit (Thermo Fisher, US). RNA-sequencing library preparation was done using Illumina TruSeq ribo-zero gold kit following manufacturer’s instructions. For each sample, 1 *μg* of total RNA was used for sequencing library preparation. Quantification of prepared libraries was performed using a Kapa quantification kit (Kapa systems) with a ABI7300 RT-qPCR instrument (Thermo Fisher, US). Libraries were further normalized to ensure equal quantity before sequencing. Paired-end, 2 × 150 bp, reads were obtained using an Illumina NextSeq 500 instrument with 300 high-output kit.

### Mapping of RNA sequencing raw reads and differential gene expression analysis

Quality of raw reads were checked using FastQC (https://www.bioinformatics.babraham.ac.uk/projects/fastqc/). Before sequence alignment, sequencing raw reads were first filtered to remove those shorter than 50 bp. For sequence alignment, the genome reference and annotation file of *Bos taurus* (NCBI, UMD3.1) were downloaded from Tophat website (http://ccb.jhu.edu/software/tophat/igenomes.shtml) and used as reference. Raw reads from all whole transcriptome RNA-seq libraries were aligned to the *B*. *taurus* reference genome using a two-step alignment approach. First, Tophat2^98^ was used with the following settings: ‘-r 70–mate-std-dec 90′ for paired-end reads from Illumina RNA-seq, Second, unmapped reads from step one were realigned with Bowtie2^99^ using the “–very-sensitive-local” method. Raw read counts for each gene were obtained using HTSeq (v0.6) HTseq^100^. Combined (Tophat + bowtie2) sequence alignment generated by the two-step alignment approach served as input file for HTSeq. The expression level of mRNAs in each sample were normalized to FPKM using cufflinks^101^. FPKM values calculated by cufflinks^101^ were used to assess gene expression profiles for each sample. Total number of expressed genes were calculated using a FPKM cutoff value of 1.

Differential expressed gene (DEG) analysis between the Treated and Control groups was performed using R/Bioconductor package DESeq2^102^ with raw read counts calculated by HTseq. Read count normalization was performed using the regularized logarithm (rlog) method provided in DESeq2. Genes with less than ten normalized, mean read counts were excluded from further differential expression analysis. False discovery rate of 0.1 was used when performing differential gene expression analysis. DEGs were determined by adjusted *p-*value corrected by Benjamin-Hochberg method (cutoff of 0.05) and the fold change (cutoff of 1.5) by DESeq2. Gene function annotation and pathway analysis were performed using DAVID^103^ and stringDB^104,105^. The top 1% most highly expressed genes were first identified for each sample using FPKM values. Then, for both treated and control groups, the shared, most abundantly expressed genes were identified. The list of the most highly expressed genes that are unique to treated group was also identified.

### Taxonomic classification of rumen wall microbial community using rRNA-sequencing reads

RNA-sequencing reads used for rumen epimural microbial community classification were done using the microbial rRNA reads generated by RNA-sequencing of rumen papilla tissues. After total RNA extraction from rumen papillae, RNA-sequencing library preparation was done using Illumina TruSeq ribozero gold kit following manufacturer’s instruction using 1*μg* of total RNA. The steps illustrated in Supplemental Fig. 1 were used for microbial community classification analysis. In brief, using STAR as the alignment tool^106^, RNA-seq raw reads mapped to the genome of *Bos taurus* (NCBI, UMD 3.1) were first filtered out. To enrich reads coming from microbial rRNA, the remaining, non-cattle RNA-seq raw reads were mapped to rRNA reference databases provided by SortMeRNA^107^ using STAR^106^. The mapped reads were used for downstream microbial taxonomic classification using Kraken^108^, following the protocol here (http://ccb.jhu.edu/software/kraken/MANUAL.html).

To compare the microbial community differences between the treated and control groups, taxonomic classifications at the genus level were considered. Genus level raw-read counts were used to identify the genera with significant abundance differences between the treated and control groups. For each sample, the total number of reads mapped to each genus level is normalized by the total number of classified reads by Kraken. To do this, the total number of reads mapped to genus level was first divided by 1,000,000, which yields the “per million” factor. Then, the mapped reads at each genus was divided by the “per million factor”, yielding a normalized read count. The statistical significance of abundance differences at the genus level abundance between the treatment groups was carried out using non-parametric test, Kruskal-Wallis, by SciPY with the *p-value* cutoff of 0.05. Genus level, expression log2 fold-change (log2FC) was calculated by comparing the average of normalized RC of treated group to that of control group. Additionally, for each treatment group, the abundance of each taxon is ranked using averaged, normalized read counts at genus level. The top 10% most common taxa were compared between the treated and control groups. Principal component analysis was performed using normalized read count at genus level with prcomp in R (version 3.2).

To assess the correlation between rumen mRNA and rumen epimural microbial rRNA expression, we performed association analysis using pearsons’r from scipy.stats (SciPy v1.2.0). The list of significantly differentially expressed genes identified using the rumen epimural sequencing data were included in the analysis. *p*-values <= 0.0001 and the absolute value of correlation coefficient more than 0.8 were considered significant. For rumen microbial rRNA expression data, normalized read-counts at genus level were included in the correlation analysis.

### RT-qPCR verification of target genes

Four randomly selected DEGs identified by RNA-sequencing were analyzed using real time quantitative PCR to confirm their differential expression between treated and control groups. These genes were: *RECQL5*, *COX20*, *PIAS* and *THUMPD3*. *RECQL5* was reported with a role in mitotic chromosome separation after cross-over events and cell cycle progress (https://www.genecards.org/cgi-bin/carddisp.pl?gene=RECQL5); *COX20* was reported as part of the mitochondrial respiratory chain complex IV^109^; *PIAS* was identified as inhibitors of the JAK-STAT pathway^110^; and *THUMPD3* belongs to the methyltransferase superfamily, and was predicted as a intracellular protein (https://www.genecards.org/cgibin/carddisp.pl?gene=RECQL5). The following Taqman probes were ordered from Thermo Fisher (Thermo Fisher, US): *RECQL5*, Bt04311476_g1; *COX20*, Bt03229764_m1; *PIAS3*, Bt03273628_m1; and *THUMPD3*, Bt03231821_ml.

cDNA synthesis was performed using 2 *μ*g of RNA with High Capacity cDNA master mix (Life technologies). All RT-qPCR reactions were performed using the ABI7500 fast system (Applied Biosystems), using gene-specific, Taqman assay probes (Thermo Fisher (Thermo Fisher, USA). The thermocycler steps were as follows: one step of

uracil-N-glycosylase (UNG)^111,112^ treatment at 50 °C for 2 min, followed by an initial denaturation/activation step at 95 °C for 10 min, then 40 cycles at 95 °C for 15 s and 60 °C for 60 s. The experiments were carried out in triplicate for each data point. The fold change in gene expression was obtained following normalization to two reference genes, Beta-actin (*ACTB)* and hydroxymethylbilane synthase **(***HMBS)*. Both of these two reference genes were found to be very consistent in the rumen epithelium^113^. The relative quantification of gene expression was determined using the 2^−ΔΔCt^ method^114^.

### Statistical analysis

Starter intake and body weight gain parameters are expressed as mean (grams) ± standard error (SE). Histograms were created for each dependent variable to confirm normality. Model fitness was confirmed by reviewing histograms of the residuals for normal distribution. Proc Mixed procedure in SAS (version 9.4) was used to analyze the following model for each dependent variable:

$${{\rm{Y}}}_{{\rm{ijkl}}}={\rm{u}}+{{\rm{a}}}_{{\rm{i}}}+{{\rm{B}}}_{{\rm{j}}}+{{\rm{y}}}_{{\rm{k}}}+{{\rm{aB}}}_{{\rm{ij}}}+{{\rm{ay}}}_{{\rm{ik}}}+{{\rm{By}}}_{{\rm{jk}}}+{{\rm{aBy}}}_{{\rm{ijk}}}+{{\rm{e}}}_{{\rm{ijkl}}}$$

where

u = the overall mean of the population

a_i_ = fixed effect of diet (A, B)

B_j_ = fixed effect of week of age (6, 8, 10, 12, 14, 16)

y_k_ = fixed effect of sampling time (−8, −4, 0, 2, 4, 8, 12, 24)

e_ijkl_ = the error term

The effect of sampling time was removed for measurements collected once per week. Either week or sampling time within week was included as a repeated effect with calf or calf within treatment as the subject. Covariance structures for each model were determined based on model convergence and Akaike’s information criterion (AIC). The slice option was used to perform partial *F*-tests to determine differences between treatments across time when significant interactions existed. Significance was declared when *p* ≤ 0.05 and a tendency when 0.05 < *p* ≤ 0.10.

## Supplementary information

Supplemental_Figure_Table

Supp_Dataset1

Supp_Dataset2

## Data Availability

Gene raw read-counts of rumen papilla samples were included in the supplemental data. rRNA raw reads rumen papilla tissues were submitted to NCBI with project accession number of PRJNA493225.
