# Supplementary material for: Transcriptome analysis of rumen epithelium and meta-transcriptome analysis of rumen epimural microbial community in young calves with feed induced acidosis
Source: Sci Rep. 2019 Mar 18;9:4744. doi: 10.1038/s41598-019-40375-2 (PMC6426933; doi:10.1038/s41598-019-40375-2)
Supplement: Supplementary file 1 — Supplemental_Figure_Table [file 41598_2019_40375_MOESM1_ESM.pdf]

Title Page: Supplemental Tables and Figures.

Transcriptome analysis of rumen epithelium and meta-transcriptome analysis of rumen epimural microbial community in young calves with feed induced acidosis

Wenli Li[1], Sonia Gelsinger[2], Andrea Edwards[1] Christina Riehle[3] and Daniel Koch[4]

[1]The Cell Wall Utilization and Biology Laboratory, US Dairy Forage Research Center, USDA ARS, Madison, WI, 53706, USA

[2]Department of Dairy Science, University of Wisconsin-Madison, Madison, WI, 53706, USA

[3]Department of Genetics, University of Wisconsin-Madison, Madison, WI, 53706, USA

[4]Department of Computer Engineering, University of Wisconsin-Madison, Madison, WI, 53706, USA

Supplemental Table 1: Summary of FPKM value of genes for all samples.

Sample Name	>=500 FPKM	>= 100 & <500 FPKM	>=15 & <100 FPKM	>0.2 & < 15 FPKM	>=1 FPKM	Total # of genes
Control1	80	230	1339	13273	11027	28538
Control2	112	209	1526	12915	11144	28538
Control3	103	227	1367	12183	10193	28538
Control4	118	212	1528	12665	10961	28538
Treat1	126	147	963	12582	9762	28538
Treat2	126	211	1657	12471	11004	28538
Treat3	99	191	1048	12226	9677	28538
Treat4	106	251	1573	12246	10580	28538

Supplemental Table 2: list of differentially expressed genes between Treated and Control groups.

Gene	baseMean	log2FoldChange (Treated vs. Control)	adjusted-pvalue
1-Mar	40.81399745	0.740943872	0.038343522
11-Sep	56.77680842	0.625397258	0.045694172
AAK1	130.1295352	-0.646315872	0.013059114
ABCA4	36.6719255	-1.294352447	0.002895701
ABCA9	478.3495894	-0.593299858	0.006818684
ABCB4	29.25153215	0.878138903	0.022301731
ABCC10	14.05041638	1.356390894	0.013527577
ABCC3	44.1790198	0.658626918	0.028811082
ABCC4	166.6922898	-1.827828653	1.75E-09
ABCC9	51.64940931	-0.662889522	0.040707606
ABHD1	66.60497025	0.588024423	0.049986967
ABHD16B	46.02734142	0.648670033	0.04632997
ABI3BP	125.7233333	0.911153012	0.002329722
ACAD11	14.35039751	1.415589271	0.013025943
ACAP3	13.89939578	-1.595073357	0.023357567

ACOT12	20.73160133	1.026337277	0.016756034
ACOX2	46.17925678	-0.956145628	0.035804893
ACPP	29.07284398	-0.853471133	0.02889681
ACSF2	18.91735294	0.887334171	0.042896997
ACSM4	14.33684752	-1.799412257	0.003313379
ACY1	26.09779086	-1.279921229	0.002730367
ADCY6	18.14094748	0.949561226	0.035249679
ADD1	42.66986337	0.963899966	0.003559223
ADH1C	15.16379108	-2.87272379	1.65E-05
ADH6	16.99366837	-2.770774997	3.85E-05
ADH6-2	26.74536089	-3.170573038	5.85E-08
ADRM1	11.64600024	1.198391677	0.030501615
AFAP1L2	25.96717067	1.801250297	0.000143602
AGTR1	864.9671419	-0.609722773	0.00135435
AHNAK2	21.39084815	1.17524874	0.005189141
AIF1	27.5938182	-0.964520787	0.013273581
AIM1	41.31930546	-0.957849452	0.008825583
AKAP5	36.15591231	1.410082393	5.98E-05
ALDH3A2	10.45291396	-1.177003573	0.04786729
ALG14	37.44351722	-1.108455193	0.003974154
ALKBH2	86.62858262	0.865763143	0.018456715
AMD1	21.20947175	1.086189836	0.035003661
AMPD3	33.33583689	1.056203083	0.010471862
ANAPC2	71.67619581	1.590618351	6.31E-05
ANGPT2	96.10107276	-0.612917788	0.035543731
ANK3	129.7766658	-0.834038618	0.00863639
ANKAR	18.68758512	1.214871495	0.017616283
ANKRD13D	25.52760819	0.857383675	0.045492272
ANKRD24	24.64323291	-4.833657279	2.12E-12
ANKRD6	58.71865455	0.678921619	0.033874598
AQP5	20.24034353	-1.227396701	0.004467834
ARG1	18.87667218	1.245320264	0.011579232
ARHGEF38	28.53746934	1.271545259	0.005873533

ART1	76.26610768	-0.640388315	0.019005774
ART1-2	15.59099928	-1.089369568	0.018007155
ASAP3	10.26326023	1.233589288	0.022277069
ASPN	23.16336688	-1.159129089	0.006702843
ATF6B	26.55376268	0.873747622	0.038675299
ATG2A	170.6961814	-0.741374484	0.01601598
ATP2C2	14.42968232	1.113692122	0.033619261
ATXN7L3	57.45100843	0.908064815	0.008473431
AUNIP	20.51857099	0.770866383	0.052730353
B3GNT5	75.55575483	-0.920807446	0.002690709
BAX	13.7233974	0.959304357	0.054447697
BBIP1	311.4223498	1.0999253	1.96E-05
BEST1	7077.410643	-0.741188501	0.000576217
BGLAP	59.17455214	0.730370641	0.043178181
BID	171.0275621	0.887732917	0.000755244
BOLA-DQA5	127.4861803	-0.771568333	0.001319317
BORA	51.2460683	0.813755612	0.00730238
BRAF	176.3098715	1.076144268	1.89E-05
BRCA2	159.4722584	1.350091027	4.30E-05
BSG	10.36175535	-1.639619765	0.00275614
C11H2orf71	21.77244287	1.520811228	0.000492546
C14H8orf37	15.31007526	-1.257864316	0.020791882
C16H1orf170	692.4991327	1.157784942	0.000617041
C21H14orf80	47.54207361	-1.283903975	0.00227738
C23H6orf201	55.75362041	-1.075645843	0.002216013
C23H6orf222	60.09641275	1.716086651	3.42E-06
C3H1orf109	71.54894709	-0.620026779	0.02779266
C3H1orf192	104.1881212	-0.698505275	0.053842816
C5H12orf23	38.8932733	-0.778322118	0.029043701
C5H12orf71	166.5739975	-0.889891674	0.000507173
C7H19orf71	18.4577239	-1.075878026	0.037688683
C9H6orf120	60.88296271	0.820835335	0.004749956
CA5B	10.95460361	-1.249201061	0.01964414

CALML4	21.45294894	-1.078665276	0.040629414
CAND2	137.4176191	0.637107356	0.048408829
CARNS1	37.09080026	1.25928272	0.003070768
CASC4	23.77037418	1.242931871	0.001775105
CC2D1A	13.91638946	-1.052753338	0.028931684
CCDC125	18.78846195	1.116140399	0.042985587
CCDC152	271.2611743	1.747789884	7.72E-05
CCDC30	11.9583351	1.684126573	0.00386796
CCDC38	99.01774155	0.671994707	0.052801746
CCDC39	15.50929297	1.188326678	0.01519129
CCDC80	22.97354	-2.043097572	4.41E-06
CCDC84	549.1414266	0.950793739	0.019206361
CCDC88A	42.3266209	3.750468341	0.000670444
CCNC	62.87657686	0.774074906	0.009073519
CCNH	77.01806185	0.638487183	0.045396393
CCNJ	14.11536681	-1.0526677	0.027854714
CD37	70.47701486	-0.710036051	0.02584705
CDC42SE1	12.23921449	0.988162637	0.052592929
CDIP1	73.32622361	0.645696456	0.014453976
CDK1	29.674226	-0.781227747	0.027434388
CDKL3	82.91329357	1.089190859	4.44E-05
CDNF	115.8455147	-0.683496121	0.007543649
CDSN	10.28062151	1.700695984	0.009213555
CEP290	63.1323994	0.762081431	0.014077493
CHD4	92.65321553	0.654871586	0.01566716
CHMP1B	35.18190999	-0.880129034	0.013698684
CHRNA2	45.88438152	1.033572424	0.010821294
CIB1	48.21549539	0.67914383	0.054923117
CKAP2L	170.3480618	-1.895197155	2.72E-05
CLCA1	10.96420825	1.527786661	0.032363638
CLCA3	48.44145465	1.389445149	0.003475739
CLN5	14.93998365	1.228561983	0.020708787
CNOT6	12.02814611	-0.920669324	0.052922968

COG8	45.25843701	-0.70591471	0.024299673
COL10A1	112.8790559	-0.767359086	0.00813587
COX20	524.9762007	0.975147083	0.000568903
CPD-2	37.6587636	1.082052585	0.002524288
CREB3	30.34371871	0.890822263	0.030032487
CRELD2	23.88661465	0.875095682	0.027308899
CSGALNACT1	11.52766853	2.49511641	0.000274191
CSTF3	34.83349857	0.923134788	0.009190388
CUL7	18.84777291	0.859974566	0.035872494
CYP17A1	27.7607552	1.724745438	0.000772659
DCAF15	12.3136461	1.559160985	0.003181228
DCST2	238.0696902	-0.821333404	0.006058072
DEK	15.16566114	-1.007315632	0.02391418
DENND5B	195.4312693	-1.390067595	0.000135393
DHX29	20.47362834	-1.177623423	0.004716015
DMPK	18.69492017	1.765101135	0.000163369
DNAJB12	15.45720065	-1.681713492	0.001805633
DNAJB3	240.5619491	-1.734094454	1.19E-12
DNAJC14	14.42674822	-1.212429746	0.020540891
DTNBP1	20.2399608	-1.721232475	9.74E-05
ECHDC2	20.8774837	-1.627434223	0.000120639
ECM2	21.77270086	-1.560901382	0.000868856
EGFLAM	249.7034319	-1.684536555	8.24E-09
EIF2D	19.42351058	1.088062728	0.012515032
EIF2S1	111.3482894	0.680307338	0.016590242
EMG1	496.5971553	-0.61633799	0.019127188
EMILIN3	19.08048972	1.300003182	0.002331373
ENG	49.94483184	-0.865180318	0.005096253
ENOSF1	22.22135607	-1.863841648	7.53E-06
ENTPD1	33.06949485	0.791257103	0.053787307
ENTPD2	13.38051021	1.077811522	0.030279699
ENTPD8	26.96548672	1.45554118	0.001102896
EPN3	46.92129423	-0.809284616	0.040208973

ERI2	15.85935159	-1.219203636	0.00971454
ERO1LB	35.43525609	0.750250605	0.03177886
ETV4	12.6553569	-1.058217542	0.032355869
EVA1B	176.3902563	0.721917687	0.027920884
EVI2B	48.97780032	-0.686666536	0.046131995
EVI5L	74.99538422	-0.936142618	0.001753343
EXOC7	22.51516652	2.091482563	0.000116675
F2RL2	170.4611955	-0.607194215	0.029180874
FAM131A	27.65916163	-2.568536775	8.37E-08
FAM135A	26.58535457	-0.925170326	0.034331677
FAM149B1	111.6822211	-1.348146099	2.01E-05
FAM151A	20.45955805	-1.318041279	0.014947364
FAM160A1	54.69844266	0.880504293	0.00702221
FAM166A	458.6795112	-0.868568625	0.000528505
FAM196A	147.8278305	-0.637099598	0.015124149
FAM228A	13.48212189	0.972073529	0.042700906
FAM69A	35.18024336	-1.067985096	0.013581104
FAM86A	49.08282751	1.271998601	0.000115633
FAM92B	437.9458785	1.320843284	3.26E-08
FBXO15	12.07840727	-1.129466922	0.029841434
FBXO17	55.36657915	-0.782694352	0.009792816
FCHSD2	19.68951524	0.794893801	0.052627209
FILIP1L	158.8848271	-0.774809552	0.001970209
FOXH1	23.03789077	2.072164511	9.81E-05
FRMD5	17.73958122	-1.322930726	0.007795611
FTH1	27.68998912	-0.95600457	0.00983176
FXR1	11.48141762	1.467991311	0.007012072
GIGYF1	24.6606559	0.92467668	0.041008932
GLB1L	50.0224622	0.74934221	0.016092621
GMCL1	21.07412925	1.30458371	0.021294653
GNPDA2	70.0190189	0.78650766	0.004719954
GNS	68.20976694	-1.160577894	2.65E-05
GOLIM4	34.78812257	-1.416633175	6.46E-05

GPC5	31.6655765	-0.771852379	0.021579328
GPR98	30.03696135	-2.350049816	4.84E-07
GPS2	1580.483788	-0.648624921	0.011607143
GRHL2	35.67290496	1.032820021	0.003259564
GRIN1	285.4909939	2.28417358	7.84E-10
GRM5	41.56038318	0.899899253	0.044325219
GRTP1	791.3706752	0.665332192	0.001118958
GUCY2D	67.7146259	0.741179618	0.042804879
GYG2	57.75484584	-0.676730804	0.018562159
H2AFY	160.322072	0.628964777	0.022886534
H2AFY2	26.23314707	-0.818967425	0.022319049
HAAO	12.88736313	-1.595426314	0.001801122
HDDC3	10.75157449	1.63259803	0.005660286
HEATR4	540.2958861	-1.310013184	2.63E-07
HERC6	69.60870963	0.736805174	0.027382809
HIGD2A	49.26528614	1.248620606	0.000661443
HIST1H1B	17.09678095	-1.974871104	1.26E-05
HIST1H1C	33.00440443	-1.459309248	0.001158211
HIST1H1D	10.12810206	-2.022544518	0.00064497
HIST1H1E	184.9276436	-2.65304749	1.88E-16
HIST1H1E-2	89.87664317	-1.605620109	1.01E-07
HIST1H2BN	111.2610286	-2.63178386	7.06E-12
HTR2B	109.9806036	-0.707086968	0.013412902
HYOU1	10.43694019	-1.097135277	0.051671358
IFI44L	44.76939828	-0.931255065	0.011470385
IFT140	39.25331896	0.932878145	0.024489784
IGBP1	17.19206284	-2.697254424	1.96E-06
IGLON5	23.94684244	-1.162335962	0.013280678
IL17B	83.29240251	-1.015238555	0.03276668
IL17RB	10.48566743	1.129888692	0.037809737
IL1A	139.7379832	-1.114745409	1.51E-06
IL23A	142.5181515	0.589565081	0.036830943
IL4I1	41.32216884	1.062821959	0.037340854

ILF2	22.8479404	0.84298144	0.024662244
IMP4	78.80220518	-0.677907217	0.022355488
IMPDH1	260.8172256	-2.406760756	7.51E-09
INPP5F	17.43587754	-0.91183751	0.040673984
IPCEF1	12.5946371	-1.273806904	0.009979859
IRF2BPL	20.37657008	1.55372408	0.002974797
IRF4	41.71960506	-0.710168432	0.040050628
ITGA6	45.10636007	2.201800312	3.87E-07
ITGB3	45.44587204	-0.670877358	0.041030824
ITPR1	22.71503941	0.865125507	0.050192332
ITSN2	20.93301084	0.960815029	0.037618634
IZUMO1	29.9131704	-0.972713569	0.045443546
JPH1	22.29582843	-1.034891304	0.049043746
KATNA1	30.47860261	0.780939386	0.037317521
KBTBD2	28.08559038	1.335368727	0.001086523
KCNE3	44.44472648	-1.009434268	0.010506556
KCNH1	54.00284335	1.083734081	0.000185234
KDELR3	146.8858949	0.883519669	0.000122975
KIAA0100	19.71650611	-0.842843046	0.049772992
KIAA0226L	1432.696317	-0.60508394	0.004794468
KIAA1244	27.1168887	1.61141803	0.000919478
KIAA1731	103.4201357	0.734523664	0.002733062
KIAA1755	117.1092428	0.958441206	0.010312312
KIAA2018	194.4021726	-0.688636111	0.003420085
KIF20A	49.71024529	-0.710451506	0.023259369
KIF4A	10.54817692	1.575856392	0.026055911
KRIT1	47.56281819	-0.914947709	0.003964366
KRT14	38.186547	-1.156884781	0.001682257
KRT17	215.9287599	-0.767302207	0.002480894
KRT6A	35.67062152	-0.747631325	0.040281479
LAMB2	13.21990902	1.089857182	0.029001209
LENG9	40.43714405	1.685713334	0.000449408
LGR4	17.84987613	-0.948158574	0.045710672

LIN28A	37.88697279	-0.753731048	0.027310048
LIPF	50.31870672	-2.292627878	9.90E-10
LMOD1	10.52052878	-2.011381311	0.012087911
LOC100137800	47.51588621	0.787359603	0.045819173
LOC100138235	91.64890676	-0.798620328	0.008697477
LOC100138660	127.1331193	-1.021616193	0.004110339
LOC100139164	68.79374815	-0.843440489	0.002853449
LOC100140121	29.44597353	-2.763412975	1.58E-09
LOC100295554	12.62299441	4.215624199	8.97E-08
LOC100295848	20.23621369	1.139987677	0.009772015
LOC100298923	228.8063853	-0.611677844	0.005710002
LOC100300956	166.8299725	0.607289832	0.032964049
LOC100335263	60.5128523	0.9003406	0.001725406
LOC100335716	64.41884408	0.641292486	0.041164306
LOC100337090	15.32478644	1.3321466	0.015974132
LOC100847171	17.37636543	-1.149159287	0.031262553
LOC100847423	51.82521273	-1.328759143	3.30E-05
LOC100847505	25.83753931	0.839920706	0.043549741
LOC100847604	17.7059276	-1.197288179	0.009757774
LOC100847716	14.80216137	-1.090324236	0.019873172
LOC100847780	70.5950619	0.681491272	0.026548491
LOC100847791	14.51485636	-1.248722562	0.040335744
LOC100847870	431.3092829	-1.446196281	8.04E-05
LOC100847874	399.875039	-2.047618022	3.01E-11
LOC100847886	14.49635772	-1.191148777	0.031571377
LOC100848100	43.16662062	2.381227788	1.02E-06
LOC100848407	226.1178627	-0.680956049	0.003882142
LOC100848495	10.04231176	-1.716812592	0.002361582
LOC100848533	19.66623397	-1.746750934	0.001143271
LOC100848660	145.9732307	-1.214061771	0.010343067
LOC100848727	11.81108335	1.498063123	0.011725977
LOC100849045	49.91199682	0.871034431	0.02103081
LOC100849091	79.44229905	-0.735159253	0.013044064

LOC100849865	11.14380263	-1.253952343	0.028585093
LOC101901978	14.21007915	1.288024181	0.018544108
LOC101901998	62.75705768	-1.895318008	1.15E-07
LOC101902038	33.68805029	1.165674887	0.004850081
LOC101902257	17.10314966	1.356475808	0.021958928
LOC101902258	56.71881981	-1.398071206	8.13E-06
LOC101902418	79.46967872	-1.097269236	0.001872214
LOC101902423	11.46156657	1.290793351	0.020813483
LOC101902444	165.2037481	-1.36062328	1.03E-06
LOC101902458	244.7322344	-0.686379395	0.002911131
LOC101902497	163.7907774	-0.731181167	0.005564596
LOC101902531	77.84786134	-0.665660482	0.023852929
LOC101902645	234.7379267	1.791255309	5.61E-09
LOC101902723	125.209196	-0.699118577	0.015229256
LOC101902730	217.56625	0.760273755	0.0226906
LOC101902821	53.00261187	-1.18207263	0.001283398
LOC101902823	13.19295529	-3.681473091	2.26E-08
LOC101902829	26.21130755	1.063202987	0.007468574
LOC101902861	43.91723345	0.621965992	0.052274625
LOC101902867	11.76641805	1.264247134	0.024239155
LOC101902881	29.80360299	-1.589085612	0.000164604
LOC101902910	264.2659999	1.121108133	0.000666614
LOC101902922	98.09829916	1.003187142	0.001476493
LOC101902941	49.80572423	-0.68844114	0.027943063
LOC101902949	31.9997622	1.116811661	0.001571707
LOC101902950	115.4501157	0.67287721	0.009478436
LOC101903000	194.7992515	-0.666562907	0.011827647
LOC101903053	36.41475723	-0.83409423	0.037101225
LOC101903094	17.67718069	-1.273041342	0.012661632
LOC101903110	126.5979808	-0.744228263	0.003893667
LOC101903116	64.53372131	-0.647914404	0.020695827
LOC101903128	120.8273112	-1.332341843	0.000109729
LOC101903157	77.95319256	-1.191527522	5.26E-05

LOC101903216	44.55769219	1.310897797	5.90E-05
LOC101903225	32.3975187	-0.863266502	0.021222328
LOC101903292	21.41879622	-1.370422943	0.008894614
LOC101903319	17.85801673	-1.145119974	0.011866967
LOC101903377	26.22776547	-0.78615996	0.045207123
LOC101903388	58.07457438	-0.842334951	0.003118974
LOC101903400	322.0058533	-0.601776918	0.040696648
LOC101903424	18.67886508	2.153395106	0.001321015
LOC101903503	19.9236868	1.282195818	0.00967115
LOC101903620	35.80137849	1.042004129	0.005213126
LOC101903680	18.52614943	-1.016668776	0.033452341
LOC101903695	18.39139598	1.040705538	0.017870608
LOC101903720	207.096744	1.179603193	0.002031176
LOC101903767	17.2092777	-1.991137154	2.30E-05
LOC101903803	40.00183476	-0.738756555	0.033513928
LOC101903835	98.63371169	-0.806059642	0.002807352
LOC101903879	75.85582101	-1.053223053	0.000120408
LOC101903908	20.33995714	-3.354851254	1.92E-07
LOC101903968	17.91419219	-1.077425958	0.012699611
LOC101903991	20.18090918	2.878777712	1.89E-07
LOC101904046	64.57632579	-0.786940916	0.0053719
LOC101904062	38.70589799	-0.910821631	0.004234789
LOC101904172	40.29843069	-0.918442848	0.00328465
LOC101904185	10.77969033	1.362086093	0.027533492
LOC101904206	10.51570709	-1.725289502	0.004186335
LOC101904248	239.2903831	0.829538513	0.006380119
LOC101904277	64.92265684	-0.728990907	0.044775596
LOC101904290	13.90015378	1.7754469	0.000581666
LOC101904321	21.80658562	-1.281291057	0.002482057
LOC101904326	28.05666347	-0.872055613	0.049785694
LOC101904456	80.42180549	0.784401974	0.002457715
LOC101904519	57.37416135	-0.991550929	0.003002798
LOC101904557	93.90112679	0.622304101	0.050439227

LOC101904572	10.39527049	-1.450112601	0.035906522
LOC101904586	205.4187193	0.729447761	0.00345606
LOC101904677	193.7096575	-1.063734587	0.001471124
LOC101904727	468.6643058	-1.031908219	0.000110788
LOC101904740	44.49554176	-0.881512341	0.009180585
LOC101904805	77.11112307	-1.38180977	7.14E-05
LOC101904832	131368.8857	1.973550437	0.041218205
LOC101904902	104.7719654	0.590860553	0.018273724
LOC101904968	66.58709249	-0.638049031	0.023347321
LOC101904988	49.98734949	-1.423984773	3.16E-05
LOC101904989	229.7977227	-0.651951331	0.006017596
LOC101905012	94.99090048	-1.494378023	0.001194608
LOC101905028	17.14915392	2.206277318	0.000178493
LOC101905173	112.1524801	-0.961766369	7.49E-05
LOC101905299	10.52754414	1.179894151	0.054052948
LOC101905310	40.39105729	-0.856603065	0.008218654
LOC101905415	114.5447912	-1.009957885	0.000721996
LOC101905455	801.7619217	-1.116356009	9.47E-09
LOC101905524	14.82380698	-0.929923642	0.052134905
LOC101905573	55.90001022	-0.686943743	0.037154146
LOC101905591	29.50009829	-1.259781349	0.000742323
LOC101905649	11.46976061	1.474287482	0.017735637
LOC101905674	22.36104872	-1.060580677	0.033398558
LOC101905676	45.7145787	-0.753873358	0.02778471
LOC101905708	16.07305074	-2.871137301	4.89E-06
LOC101905714	11.64293229	-1.650038855	0.00531577
LOC101905737	45.02078819	-0.855447007	0.013099071
LOC101905792	357.6958043	-0.913001765	0.001316166
LOC101905851	137.7812975	-0.871761959	0.000261498
LOC101905920	160.3571353	2.76520657	6.89E-05
LOC101905947	58.80052903	-0.802843407	0.023981497
LOC101906031	94.49756006	-0.613573982	0.014524841
LOC101906076	39.36946178	-0.837142639	0.013935578

LOC101906215	173.418533	-0.642004357	0.009283405
LOC101906431	40.40126711	-1.029218984	0.003893513
LOC101906469	55.78688891	-1.279167649	0.015023633
LOC101906563	123.4513343	-0.918402164	0.002541057
LOC101906586	33.91114152	-0.816769961	0.044113147
LOC101906640	87.268791	-0.855996925	0.025850566
LOC101906656	15.02372835	-1.179579915	0.031037737
LOC101906810	35.98598388	0.718831177	0.0288127
LOC101906924	151.7540044	-1.151501625	3.49E-05
LOC101907068	27.2533338	1.00513581	0.008163061
LOC101907239	165.162267	-0.627773162	0.004271762
LOC101907276	140.6488444	-1.832546569	4.58E-09
LOC101907288	445.4705929	-0.593921684	0.003255174
LOC101907548	14.36978381	1.8817271	0.000680897
LOC101907617	16.72688091	-2.26205212	1.83E-05
LOC101907618	142.0078625	0.843759293	0.000473585
LOC101907799	37.78177728	-1.130959416	0.009296101
LOC101907999	18.78961799	1.172052467	0.008300149
LOC101908002	118.7295274	1.360521862	4.93E-07
LOC101908009	161.198638	-0.629079906	0.004560086
LOC101908154	176.1077841	-0.669729245	0.008096774
LOC101908170	597.2343313	-1.16941206	7.13E-07
LOC101908207	107.1872822	0.762586686	0.015438877
LOC101908232	11.72628554	-1.412800568	0.00614089
LOC508933	26.50906339	0.969987783	0.022709652
LOC515517	100.8089473	0.856055312	0.036457415
LOC515578	17.86587157	1.141978533	0.01261799
LOC518907	318.5455646	-0.642030329	0.003277263
LOC519492	38.56668802	1.07831207	0.007722931
LOC521083	21.25800589	-1.158840407	0.011850393
LOC522540	13.05491266	-1.152835821	0.015977203
LOC529277	348.9300179	-2.476510789	7.55E-23
LOC530613	227.7946341	-0.629778837	0.008499768

LOC530739	498.4651126	-0.617169631	0.007615093
LOC537848	12.49901885	-1.572297017	0.015041454
LOC539106	64.4444415	0.731697244	0.018721086
LOC540148	510.1657501	-2.137353252	7.95E-16
LOC540863	309.0064283	-0.603752835	0.019277573
LOC613595	14.4489732	-1.043114719	0.031925861
LOC613677	74.13618513	-0.875564389	0.003704987
LOC613715	47.4516118	0.724109633	0.016881678
LOC614614	14.75556897	1.182266	0.01341776
LOC615257	208.3705613	-0.655616684	0.003510405
LOC616819	1068.13151	-1.539409352	5.34E-10
LOC616853	10.9022754	-1.572157001	0.009190437
LOC617905	3986.998322	-1.391585037	3.04E-06
LOC781253	13.60174745	-1.430791991	0.004119467
LOC782021	32.69173823	-0.859247053	0.013848003
LOC782525	13.16035738	1.440199466	0.004550456
LOC784451	78.34317658	-0.659480541	0.019145145
LOC784768	97.97857116	1.485336543	0.000322569
LOC784939	32.61797525	-3.367746098	1.66E-14
LOC785150	77.76463608	-0.631926323	0.048467682
LOC786060	93.17462387	-0.59410561	0.030134573
LOC787269	10.54507	-2.336123259	8.22E-05
LOC788530	20.034417	0.90923105	0.047374366
LOC789362	189.9166363	0.59247069	0.021629624
LPAR3	66.18905188	-0.808503463	0.004451778
LPCAT3	269.0827784	-0.604160851	0.028758831
LRRC41	40.60320093	-1.297737284	0.000203022
LRRC41-2	45.86840072	-1.499256437	1.42E-06
LRRC47	13.72822207	-1.22722346	0.013017064
LRRC57	52.12462363	0.711011603	0.021650628
LUZP1	98.96919335	-0.951121065	0.001135713
LY6G6F	19.93824208	1.455192164	0.007920402
LYSMD2	36.68791132	-0.837154535	0.018839073

LYZ	29.27050209	-0.854475599	0.034424435
MAFK	18.10848285	1.001356787	0.042635745
MANEAL	14.6565772	1.222271111	0.014690071
MAP3K6	30.29458174	0.985388878	0.007272058
MARS	13.40735533	1.408799312	0.007389378
MASP2	270.0475751	0.591846567	0.009237041
MCCD1	30.09043232	0.719966669	0.041679828
MCOLN1	20.07481212	-0.939406299	0.024847104
MFSD6	94.19960278	0.816197451	0.003845437
MGC151921	11.05529057	-1.469960225	0.022069372
MICAL1	23.9962972	0.727720679	0.050140653
MIEN1	113.1439393	0.903782199	0.003092566
MIR181A-2	31.01259203	-0.799370062	0.019184372
MIR181B-2	29.96189913	-1.295357592	0.004169892
MIR425	19.75760909	1.623600519	0.000627792
MIS18BP1	26.63802198	0.922979488	0.012599178
MPO	13.79985809	6.406771309	1.63E-08
MRO	238.2297911	-1.721270578	5.34E-08
MROH8	131.5586056	-0.809549889	0.002290446
MRPL49	19.44303901	0.890072381	0.039773454
MRPL53	28.21646932	1.318833862	0.000471046
MRPS18C	10.85179508	1.386084677	0.015098337
MSH3	13.47933503	1.149119531	0.030041156
MTF2	46.89897133	1.028477558	0.004259484
MTIF2	8667.150707	0.709901413	0.03123597
MXRA8	50.84244012	0.695362758	0.051317381
MYH10	131.8694349	-0.626277391	0.006403049
MYH9	33.00781755	-1.040966166	0.007370444
MYOF	77.93099867	-1.788040793	3.73E-07
NAP1L5	48.26127966	-0.617798137	0.04889977
NAPSA	13.07531967	-1.407336272	0.01374737
NCMAP	116.613689	-1.363712288	1.34E-06
NDUFB8	18.36017305	-0.921509213	0.039260447

NDUFB9	142.4527143	0.695850542	0.037397922
NEMF	49.34516923	1.115347518	0.001874189
NFAT5	31.93344898	-0.692118508	0.05283381
NFATC3	378.3290844	0.884815051	0.000222483
NHSL2	26.1510759	-3.017212186	3.41E-08
NME7	23.37763259	1.353059488	0.003474671
NMT1	13.3592506	2.069385878	7.90E-05
NMT2	58.11373183	-0.676554118	0.045299098
NO66	101.1526527	-1.50913797	1.07E-07
NOS3	69.64260794	1.034340558	0.002614236
NPHP3	12.71165376	1.225916178	0.02178002
NPL	61.21534338	-0.676560539	0.020252207
NRM	49.63733284	-1.269846051	0.004030466
NSD1	148.3492722	1.34097054	1.98E-06
NSMF	64.06688046	0.947674541	0.035925809
NT5DC2	33.24244165	0.879067505	0.019091967
NT5DC4	50.61747922	-2.134277986	3.28E-07
NUDT5	11.69791172	1.222174653	0.054304332
NUMB	66.18952804	0.906619321	0.002979712
NUP85	84.12608737	0.784108947	0.004766774
OLFML2A	14.21628423	-1.294569078	0.005514698
OMD	35.83035584	-1.391715137	0.001090052
OXER1	22.24649756	-0.95249163	0.04201508
P2RX5	34.16639946	0.893097891	0.007109755
PACS2	29.55911424	-0.883198074	0.023105151
PAIP2	16.63100334	1.628104024	0.000714141
PARD6A	50.59491016	0.672639649	0.038961283
PCSK7	462.4874129	-1.411697107	0.001435624
PDHB	23.17278423	-1.042374708	0.008754474
PEX3	61.79078209	0.608226281	0.054246046
PHC3	137.0419392	0.903836247	0.000240167
PIAS3	173.8306152	1.21594523	6.08E-05
PIGF	57.21335312	0.761177509	0.047009625

PIGM	41.05439613	-0.651464688	0.039007142
PIK3CA	23.09923901	0.88779941	0.024957362
PIK3CD	14.02658529	1.581017636	0.001678432
PIK3IP1	30.23537516	1.145089407	0.009089101
PIK3R1	156.4936898	-1.273111281	0.000171532
PINX1	18.37710559	1.079544887	0.032246971
PITPNM1	17.27283057	0.998450346	0.028425237
PKD2L2	232.7290691	0.613926906	0.013193422
PLCXD2	16.08806467	-1.912856484	0.000251537
PLEKHG7	166.7493642	0.97031613	0.000372197
PMF1	36.59681744	0.73857136	0.031041583
PMVK	10.85298069	-1.345755064	0.048524783
PODNL1	140.4893103	0.731994463	0.027600904
POLG	20.3186758	-1.274150795	0.010707462
POLR2B	116.4571392	0.984561864	0.019318413
POLR3GL	53.47308906	-0.71645413	0.023237831
PPARG	14.02919325	-1.130072066	0.018473415
PPP1R11	24.10036139	1.025629248	0.023972578
PPP1R12C	42.93620963	1.156199518	0.017348196
PPP1R16A	242.4155087	0.777659164	0.031796138
PPT2	29.31258612	1.284498108	0.010789839
PRKAR1A	11.95353575	2.342500435	0.000301254
PROX2	48.99918048	1.380372692	5.33E-06
PTOV1	41.21761121	0.98097824	0.018382271
PUF60	33.26241491	1.59007551	0.00053456
PYROXD1	44.67574925	0.603059145	0.05276292
QRSL1	29.7402003	1.150686962	0.037120115
RAB14	41.93169221	0.819113813	0.010212999
RAB19	22.67814109	-0.926321255	0.016034068
RAB21	18.39427792	0.840310874	0.051501397
RABL6	45.88093553	-0.851438617	0.00818304
RAPGEF4	50.13656501	-1.154517727	0.011373767
RAVER2	192.9843675	0.914489693	0.014895511

RBM4B	56.61704322	-0.712236258	0.035019225
RBPJL	14.91110229	1.988705247	0.003323667
RECQL5	219.7803996	1.600685639	6.06E-05
REP15	29.27437358	-0.798359752	0.033083324
RIPPLY3	69.93736763	-0.62545159	0.022177203
RMDN3	41.36927082	-1.164286543	0.001352055
RNFT1	12.27055201	1.114685984	0.040680034
RPE	35.72643782	1.687220562	1.76E-06
RPL18	21.56353651	0.903596155	0.028870172
RPLP1	20.4319916	0.776486	0.051674835
RPS24	30.57304551	0.973866391	0.021519217
RPS27L	16.73574285	0.890699735	0.041747697
RRP7A	12.36230353	-1.813897829	0.003943895
RTDR1	12.19065391	-3.4599418	1.05E-05
RUFY1	140.5267797	-0.813268907	0.000533833
RUNX2	19.80679617	-0.888107158	0.030680216
SCNN1G	21.85879932	-0.896188977	0.017297564
SCRN3	65.62313053	0.762824347	0.024839191
SCYL3	13.19170197	-1.543569593	0.009183914
SECISBP2	16.23511871	1.924765558	0.000176775
SEMA3C	20.08846956	-5.166181275	6.04E-10
SERINC4	376.7086898	0.749326731	0.001630842
SETD1A	22.44460358	0.81727467	0.050188953
SETD2	19.19854045	0.880817798	0.050603971
SIAE	10.83731278	1.638704221	0.012059281
SIGIRR	50.59956936	0.649516996	0.042055159
SIRT4	3016.46997	-0.961084484	0.000459756
SLC12A6	74.44171853	1.009401276	0.009179347
SLC15A2	35.64777001	-0.908896946	0.006291691
SLC16A1	18.0493968	-1.052922786	0.015464269
SLC22A23	26.36136147	1.768711957	5.97E-05
SLC25A10	17.27625156	1.358912398	0.020865416
SLC25A42	204.4824732	1.02890236	0.001124622

SLC30A4	149.6399769	-0.787935091	0.003080485
SLC31A2	62.37144396	0.590888749	0.025609826
SLC9A3	77.39882446	-0.778523422	0.047463846
SLK	83.30106475	1.205728391	1.32E-05
SMARCD3	24.81605201	1.045019838	0.043605337
SMIM17	24.27971699	0.948849428	0.025210284
SNRPD3	33.08432152	0.759818478	0.048378594
SNX13	46.32774707	1.171267413	0.000679868
SNX21	352.6360545	-0.961916502	0.005556516
SNX32	986.4028334	-0.838337465	0.000603982
SPHK2	11.95309687	1.066869077	0.04883808
SPINK7	24.40361006	0.941307805	0.034938332
SPINK9	36.18068682	1.493521369	0.004235577
SPTBN5	31.4837491	1.451179834	0.000182528
SRRT	30.28363989	0.824208624	0.023032018
SRSF12	24.69218008	0.750490453	0.054913484
SSH3	70.60659829	0.89624074	0.020246497
SSLP1	76.43777034	-0.624735724	0.013501064
ST6GAL1	68.61519705	-0.808278133	0.005231307
STAT4	47.08076568	1.270359521	0.000303917
STIP1	15.5356809	-1.347338218	0.00660107
STX4	38.54014191	0.833588449	0.014548122
STXBP5L	98.45938677	-2.27026558	8.61E-10
SURF4	11.70210784	1.12530134	0.04516908
SYN1	12.85362483	1.108639984	0.041116484
SYNCRIP	28.93678641	1.938693206	4.20E-05
SYNE1	16.77509451	1.202164456	0.035853918
SYNRG	23.21864987	1.70314469	0.000166129
TAF8	88.4204045	1.310740932	0.0002437
TAGAP	14.18108472	1.311174034	0.006889416
TAX1BP3	76.10801029	0.669589782	0.03340864
TCHH	68.36674371	-3.256974276	7.73E-15
TCP1	513.5006237	-0.594055889	0.016213132

THADA	10.01348844	1.691122577	0.003023456
THUMPD3	218.7470161	0.928527863	0.000369498
TIMM22	46.007764	0.986441264	0.009965325
TMC2	10.12370173	-1.665100273	0.009303167
TMEM120B	84.35640915	0.743691861	0.024143727
TMEM150A	307.4160357	0.79534879	0.007859246
TMEM161A	301.2695141	-1.026778798	0.00152142
TMEM173	10.46130591	-1.139518527	0.042557549
TMEM201	23.52981934	-1.00187078	0.012961182
TMEM223	18.70166577	0.998262291	0.015616902
TMEM79	84.50494484	0.632247241	0.01982309
TNXB	23.88758959	-1.216361467	0.002696439
TOMM40L	14.81189826	2.051879354	0.001175564
TOP1	41.23303404	0.746974593	0.024922792
TOR1AIP1	33.41138151	-1.217542618	0.001316511
TP53INP1	44.88061191	-0.738446301	0.033368816
TP53TG5	34.25044029	-1.058607191	0.004363298
TPM4	10.03209838	-1.312431015	0.027962903
TPMT	22.42097867	-1.092598218	0.016774651
TRAF3IP3	81.50077506	-0.882669992	0.00234068
TRMT5	28.45438085	-0.845487746	0.03646979
TRNAC-ACA-64	13.7000724	-1.075522854	0.027882238
TRNAC-GCA-169	12.21538997	-1.479946807	0.003836681
TRNAE-UUC-55	26.95762684	-0.944311293	0.014256259
TRNAL-CAA-5	39.32264573	-0.725258957	0.031691338
TRNAR-UCU-11	10.32700251	1.51057047	0.014957101
TRNT1	90.02396766	0.822004904	0.001509677
TRPV2	16.61713656	-0.880258923	0.049871714
TRPV6	24.6305753	3.928869837	1.33E-12
TSPAN14	43.33955511	1.016141494	0.025534911
TTC1	20.21382375	0.956384522	0.020570747

TTC23	11.31923574	-1.809361507	0.03173893
UGGT2	10.28625659	1.060448707	0.051887793
UGP2	29.83830936	0.935597816	0.049832128
UHRF1	65.6843035	0.845024478	0.008261243
UPP2	173.8814209	-0.583844484	0.036813987
USP53	33.89574395	1.034011335	0.010304652
USP8	23.98349337	0.759083379	0.038468974
VLDLR	39.76158993	-0.891177341	0.014196062
VSIG8	23.04870957	1.223598429	0.006574158
VWA2	89.95184645	0.611335729	0.010857118
VWA8	10.73018178	-1.491089611	0.00892794
WBP11	11.99975825	1.228258839	0.031220015
WDFY2	10.46944102	1.427255043	0.016622048
WDR3	36.13796084	-1.152690473	0.000974685
WDR37	36.04581336	0.971455868	0.004864138
WIPF1	29.86746554	-0.825133581	0.031836046
ZCCHC14	42.90681972	0.612938025	0.050151908
ZDHHC20	22.54172328	1.237986722	0.003742245
ZDHHC3	20.03764387	-1.69095866	0.001189231
ZFHX3	37.61527731	-1.709230239	1.29E-06
ZFP30	264.7520139	-0.592691854	0.02162877
ZFP41	165.6527623	-0.609290432	0.036286855
ZHX3	77.57425666	0.626936526	0.052867998
ZMYM4	18.00341858	1.087095644	0.027594226
ZNF143	17.18984146	-1.041833085	0.015600463
ZNF219	106.6351016	-1.06392839	0.00041508
ZNF276	67.80109478	0.778757816	0.020384255
ZNF277	31.6662286	-0.806445076	0.029626344
ZNF397	17.34804485	-1.140010487	0.028849791
ZNF615	17.34785783	-0.878620946	0.051739086
ZNF845	17.23819812	0.998846026	0.026938221
ZXDC	66.58634816	0.638929073	0.035154628

Supplemental Table 3: List of genera with increased abundance in Treated group.

Genus	Phylum	Gram negative or positive	BaseMean	log2FC (Treated vs. Control)	p-value
Comamonas	Proteobacteria	Gram negative	39992.77	6.72	4.44E-12
Rickettsia	Proteobacteria	Gram negative	107.31	6.57	4.48E-07
Actinobacillus	Proteobacteria	Gram negative	122.01	3.06	2.62E-05
Salinispora	Actinobacteria	Gram positive	512.93	3.33	8.44E-05
Olsenella	Actinobacteria	Gram positive	35964.69	3.28	0.00012011
Methanosarcina	Euryarchaeota	Archea	5789.69	11.89	0.00019007
Aeromonas	Proteobacteria	Gram negative	221.24	3.91	0.00164748
Desulfovibrio	Proteobacteria	Gram negative	12908.70	2.10	0.0041795
Brevibacterium	Actinobacteria	Gran positive	200.55	2.92	0.00456062
Fusobacterium	Fusobacteria	Gram negative	1663.29	2.53	0.00765833
Tannerella	Bacteroidetes	Gram negative	768.69	2.28	0.00792209
Porphyromonas	Bacteroidetes	Gram negative	322.33	2.37	0.00813521
Azoarcus	Proteobacteria	Gram negative	1490.78	4.02	0.01342644
Brevibacillus	Firmicutes	Gram positive	766.65	2.14	0.01584166

Supplemental Table 4: List of genera with decreased abundance in Treated group.

Genus	Phyla	Gram negative or positive	BaseMean	log2FC (Treated vs. Control)	adjusted pvalue
Mycobacterium	Actinobacteria	has both	581.643086	-5.0627258	3.03E-06
Bifidobacterium	Actinobacteria	gram positive	1062.28578	-3.1683983	0.00017443
Lactobacillus	Firmicutes	gram positive	13437.5779	-2.335366	0.00166069
Cyanothece	Eubacteria		261.061579	-1.9598666	0.0214063
Gloeobacter	Cyanobacteria		187.01674	-1.4337154	0.04037755

Supplemental Table 5: significant correlation between mRNA and the rRNA (at genus level).

Gene Name	Genus Name	Correlation Coefficient	p-value
KCNE3	Gemmatimonas	>0.9	<0.0001
CASC4	Actinobacillus	>0.9	<0.0001

MIEN1	Mannheimia, Delftia	>0.9	<0.0001
SLC22A23	Actinobacillus, Delftia	>0.9	<0.0001
FAM151A	Gordonia	>0.9	<0.0001
TMEM223	Salinispora, Agrobacterium, Novosphingobium, Xanthomonas, Sinorhizobium	>0.9	<0.0001
GRIN1	Actinobacillus	>0.9	<0.0001
ENTPD2	Actinobacillus	>0.9	<0.0001
PCSK7	Gordonia	>0.9	<0.0001
GRM5	Methanosarcina, Agrobacterium, Novosphingobium, Xanthomonas, Sinorhizobium	>0.9	<0.0001
ABCC10	Hymenobacter	>0.9	<0.0001
ADCY6	Aeromicrobium	>0.9	<0.0001
STX4	Actinobacillus	>0.9	<0.0001
ENTPD8	Actinobacillus	>0.9	<0.0001
ENTPD8	Actinobacillus	>0.9	<0.0001

Supplemental Figure 1: Analysis steps for microbial community classification analysis. STAR is used as the alignment tool for this workflow. RNA-seq raw reads mapped to the genome of *Bos taurus* (NCBI, UMD 3.1) were first filtered out. To enrich reads coming from microbial rRNA, the remaining, non-cattle RNA-seq raw reads were mapped to rRNA reference databases. The mapped reads were used for downstream microbial taxonomic classification using Kraken.

Supplemental Figure 1

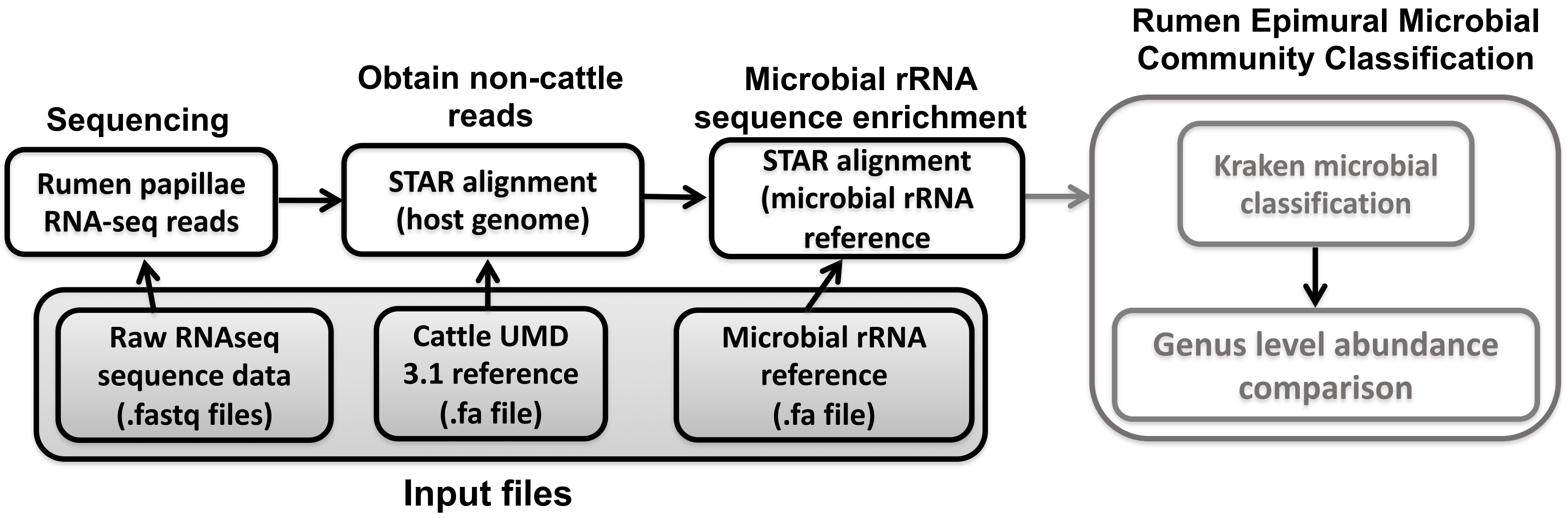

Supplemental data 1: ph values of all calves in the study at all sampling points.

Supplemental data 2: Gene raw read counts for samples in Control and Treated groups.
